# The Effect of Thymoquinone on Acoustic Trauma-Induced Hearing Loss in Rats

**DOI:** 10.7759/cureus.72181

**Published:** 2024-10-23

**Authors:** Mustafa Said Tekin, Abdullah Ayçiçek, Abdulkadir Bucak, Şahin Ulu, Erdoğan Okur

**Affiliations:** 1 Otolaryngology - Head and Neck Surgery, Medipol University Medipol Mega Hospital, Istanbul, TUR; 2 Otolaryngology - Head and Neck Surgery, Afyonkarahisar Health Sciences University Hospital, Afyonkarahisar, TUR; 3 Otolaryngology - Head and Neck Surgery, Suleyman Demirel University, Isparta, TUR

**Keywords:** acoustic trauma, antioxidant therapy, otoacoustic emissions, oxidative stress, sensorineural hearing loss, thymoquinone

## Abstract

Background

The aim of this study was to investigate the protective effect of thymoquinone, an antioxidant, on hearing loss induced by acoustic trauma in rats.

Material and methods

This study included 32 Wistar Albino rats divided into four groups: control, acoustic trauma, thymoquinone + acoustic trauma, and thymoquinone only, with eight rats per group. The control group received 0.5 mL of corn oil intraperitoneally for 10 days. The acoustic trauma group was exposed to 100 dB white noise at 4 kHz for 16 hours. The thymoquinone + acoustic trauma group received thymoquinone (10 mg/kg) intraperitoneally for two days before acoustic trauma and eight days after acoustic trauma. The thymoquinone only group received thymoquinone (10 mg/kg) for 10 days. Distortion product otoacoustic emissions (DPOAEs) were measured before and after treatments on days 1, 4, and 10.

Results

In the control group, DPOAE measurements showed no significant change over the study period. The acoustic trauma group exhibited a significant decrease in DPOAE on the first day after trauma, followed by some recovery. The thymoquinone + acoustic trauma group showed no significant decrease in DPOAE on the first day post-trauma, suggesting a protective effect. The thymoquinone only group also indicated no significant change in DPOAE measurements, suggesting that thymoquinone alone did not affect hearing function.

Conclusion

Thymoquinone demonstrated a protective effect against acoustic trauma-induced hearing loss in rats, as evidenced by stable DPOAE measurements post-trauma. These findings suggest that thymoquinone may help preserve hearing function by reducing oxidative stress in the cochlea. Further studies are needed to confirm these results in humans and optimize dosage and treatment protocols.

## Introduction

Acoustic trauma, one of the common causes of sensorineural hearing loss, is defined as the direct damage of noise above 85 dB to the outer hair cells of the inner ear and the supporting cells of the organ of Corti [1-3]. Although many mechanisms have been proposed for noise-induced hearing loss, the pathogenesis of this condition is still not fully understood. The main trigger of hearing loss is apoptosis in the organ of Corti [4]. As a result of acoustic trauma, factors such as decreased blood flow to the inner ear, lack of oxygen, and increased reactive oxygen species due to increased metabolic activity lead to cell death. This process is triggered by the energy of high sound waves and reduces cochlear blood flow by disrupting microcirculation, altering cellular DNA structure, and damaging the cell membrane through lipid peroxidation. This leads to cellular necrosis and overall noise-induced hearing loss [5,6].

Various antioxidants have been studied to alleviate oxidative stress in the cochlea to reduce hearing loss caused by acoustic trauma [7,8]. One study revealed that antioxidants with free radical scavenging properties may reduce noise-induced hearing loss [9]. Nigella sativa is popularly known as black cumin seed, and the main component of this plant is thymoquinone. Thymoquinone has many effects such as antidiabetic, antioxidant, antihistamine, anti-inflammatory, antimicrobial, antitumor, antiaggregant, and immunomodulatory. It also exhibits antioxidant effects by scavenging superoxide radical anions and hydroxyl radicals [10]. Thymoquinone has been found to prevent oxidative damage in hepatocytes by inhibiting superoxide dismutase, catalase, and glutathione peroxidase enzymes [11]. These effects are generally realized by suppressing cyclooxygenase and lipoxygenase enzymes and inhibiting membrane lipid peroxidation [12].

Otoacoustic emissions (OAEs) are low-intensity, nonlinear acoustic signals produced in cochlear vibrating hair cells at the perineural level, independent of afferent neural integration [13]. OAEs fall into four main categories: spontaneous otoacoustic emission, transient evoked otoacoustic emission, stimulus-frequency otoacoustic emission, and distortion product otoacoustic emission (DPOAE) [13]. DPOAE is obtained with continuous sound stimuli at two different frequencies and is used to determine damage to hair cells [14]. Signal-to-noise ratio (SNR) is defined as the ratio of the signal received from the cochlea to the internal noise recorded during the measurement. SNR values are more reliable for evaluating DPOAE responses than DPOAE amplitudes [15]. DPOAE is a highly sensitive method to detect the affected frequencies in noise-induced hearing loss and acute acoustic trauma [14].

The aim of this study was to investigate the protective effect of thymoquinone, which has antioxidant properties, on acoustic trauma-induced hearing loss in rats.

## Materials and methods

This study was conducted at the Department of Otorhinolaryngology, Faculty of Medicine, Afyon Kocatepe University, and the Experimental Animal Laboratory, Faculty of Veterinary Medicine (Ethics Committee Approval: B.30.2.AKÜ.0.A2.00.00/244). The study was performed on 40 Wistar Albino rats with an average weight of 250 g.

Otoscopic examination of all rats revealed normal external ear canals and tympanic membranes. Before the study, the rats were anesthetized with intramuscular ketamine hydrochloride (45 mg/kg) and xylocaine (5 mg/kg). Eight rats without DPOAE response were excluded from the study, and the remaining 32 rats were divided into four groups. Both ears in each group were included in the study and a total of 16 ears were evaluated. Rats were housed in a 12-hour light/12-hour dark, 25°C constant temperature environment with free access to food and water. Thymoquinone (Toronto Research Chemicals, North York, Toronto, Canada) was dissolved in corn oil and prepared for intraperitoneal injection.

There were four groups in the study. In the control group, eight rats received 0.5 mL of corn oil intraperitoneally every day for 10 days. In the acoustic trauma group, only acoustic trauma was applied to eight rats, and DPOAE measurements were performed on the pre-trauma, and post-trauma days 1, 4, and 10. In the thymoquinone + acoustic trauma group, eight rats were administered thymoquinone at a dose of 10 mg/kg every day for two days before acoustic trauma and eight days after trauma. In this group, DPOAE measurements were performed before trauma, on days 1, 4, and 10. Only eight rats in the thymoquinone group were administered thymoquinone at a dose of 10 mg/kg every day for 10 days, and DPOAE measurements were performed before and on the 4th and 10th days of administration of thymoquinone.

The acoustic trauma model was created by exposing rats to 100 dB of white noise at a frequency of 4 kHz for 16 hours. DPOAE measurements were performed using ILO 288 Echoport USB and EZ-screen 2 software. Measurements were performed after the tympanometry probe was placed in the ears and the appropriate position of the device was ensured.

DPOAEs (2f1-f2) were measured at a ratio of 1.22 f2/f1 and stimulus intensity L1=65 dB sound pressure level (SPL), L2=55 dB SPL. DPOAEs were recorded at frequencies of 1,001, 1,501, 2,002, 3,003, 4,004, 6,006, and 7,996 Hz. Each measurement lasted approximately 75 seconds. The SNR was evaluated in these frequency bands.

Statistical analyses were performed using SPSS Version 15.0 (SPSS Inc., Chicago, IL). The conformity of the variables to normal distribution was examined visually (histogram and probability plots) and analytically (Kolmogorov-Smirnov test). Descriptive analyses were presented as mean and standard deviation. Since the parameters were not normally distributed, the statistical significance of the change over time for these parameters was analyzed using Friedman's test. If necessary, pairwise comparisons were made using the Wilcoxon test. Results were considered statistically significant when p-value was <0.05.

## Results

**Figure 1:**
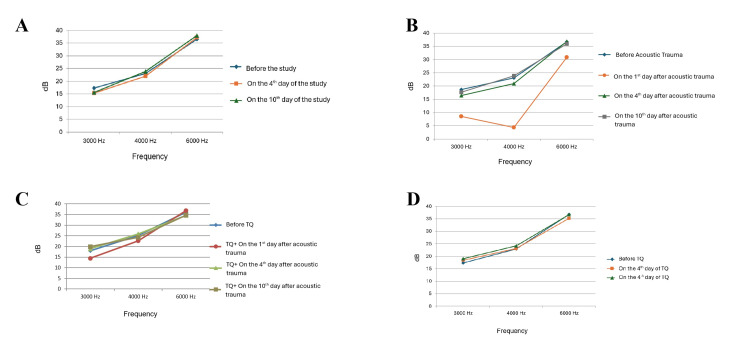
(A) OAE SNR curves of the control group according to days. (B). OAE SNR curves of acoustic trauma group according to days. (C) OAE SNR curves of TQ + acoustic trauma group according to days. (D) OAE SNR curves of TQ group according to days. OAE, otoacoustic emissions;  SNR, signal-to-noise ratio; TQ, thymoquinone

In the control group, there was no statistically significant difference between the SNR measurements made before the study, on day 4, and on day 10 (Table 1 and Figure 1).

**Table 1:** Comparison of values for four groups before the study, on day 4, and on day 10

Group	Frequency (Hz)	Before the study (mean ± SD)	Day 4 (mean ± SD)	Day 10 (mean ± SD)	Day 4 p-value	Day 10 p-value
Group 1	3,000	17.32 ± 5.52	15.27 ± 5.00	15.56 ± 6.29	0.245	0.552
	4,000	22.97 ± 3.59	22.01 ± 3.68	23.85 ± 3.90	0.469	0.485
	6,000	36.46 ± 3.42	37.21 ± 2.14	37.98 ± 3.61	0.717	0.277
Group 2	3,000	18.68 ± 4.02	16.42 ± 7.01	17.70 ± 3.51	0.423	0.140
	4,000	23.10 ± 2.35	20.93 ± 8.07	23.90 ± 2.92	0.535	0.679
	6,000	36.70 ± 2.60	36.82 ± 4.97	35.90 ± 4.21	0.796	0.649
Group 3	3,000	18.01 ± 7.09	18.89 ± 2.84	19.86 ± 3.43	0.642	0.569
	4,000	25.14 ± 4.45	25.88 ± 4.37	24.22 ± 2.70	0.756	0.408
	6,000	36.05 ± 4.69	34.52 ± 2.38	34.66 ± 2.82	0.215	0.187
Group 4	3,000	17.30 ± 4.87	18.36 ± 5.45	18.98 ± 5.34	0.501	0.469
	4,000	22.96 ± 3.80	23.17 ± 2.69	24.20 ± 4.72	0.887	0.334
	6,000	36.78 ± 3.24	35.23 ± 3.14	36.64 ± 5.31	0.170	0.856

In the acoustic trauma group, a significant decrease in SNR values was detected in OAE measurements performed on day 1 (Table 2 and Figure 1). However, there was no significant difference in SNR values in the measurements made from day 4 onwards. In the thymoquinone + acoustic trauma group, there was no statistically significant difference between the SNR measurements made on day 1 (Table 2 and Figure 1) and the measurements made on days 4 and 10 (Table 1 and Figure 1). In the thymoquinone only group, no statistically significant difference was observed between the measurements made before the study, on day 4, and on day 10 (Table 1 and Figure 1).

**Table 2:** Comparison of values for groups 2 and 3 before the study and on day 1

Group	Frequency (Hz)	Before the study (mean ± SD)	Day 1 (mean ± SD)	Day 1 p-value
Group 2	3,000	18.68 ± 4.02	8.56 ± 6.73	0.001
	4,000	23.10 ± 2.35	4.40 ± 5.48	<0.001
	6,000	36.70 ± 2.60	30.88 ± 5.74	0.003
Group 3	3,000	18.01 ± 7.09	14.41 ± 2.63	0.088
	4,000	25.14 ± 4.45	22.65 ± 7.53	0.098
	6,000	36.05 ± 4.69	36.81 ± 4.95	0.623

## Discussion

This study assessed the effects of thymoquinone treatment of hearing loss after acoustic trauma in rats. In the control group, OAE measurements did not show a significant change during the study. The absence of any variation in OAE measurements only in the thymoquinone group indicated that thymoquinone had no significant ameliorating or impairing effect on OAE measurements. In the acoustic trauma group, there was a decrease in OAE measurements on day 1, but then an improvement was observed. In the thymoquinone + acoustic trauma group, thymoquinone administration significantly attenuated post-traumatic hearing loss. In the present study, an acoustic trauma model was created by applying white noise at 4 kHz frequency and 100 dB SPL for 16 hours. In the measurements performed on the second group, statistically significant transient threshold loss was observed, confirming the effectiveness of our acoustic trauma model and the reliability of the results.

The pathophysiology of cochlear damage resulting from acoustic trauma involves two primary damage processes, namely mechanical and metabolic. High-intensity sound waves may cause significant changes in cellular systems in the cochlea, and these changes may lead to permanent or temporary shifts in the hearing threshold [16]. Particularly noteworthy are the effects on the outer hairy cells, which are the most common cause of hearing loss because of acoustic trauma. Damage such as loss of these cells, their adhesion to each other, structural fracture, and detachment from the tectorial membrane have been observed. After the detachment of the stereocilia in OHCs, a certain amount of time is required for these structures to reattach to the tectorial membrane, and this process can be considered as a cause of transient hearing loss [17]. In addition to mechanical damage, metabolic damage in the cochlea as a result of sound exposure also plays an important role. Hair cell deaths resulting from loud sound exposure are triggered by oxidative stress [18]. This further accentuates the development of processes such as increased free oxygen radicals, lipid peroxidation, and microcirculatory disorders [19, 20]. A study on the effects of acoustic trauma on the cochlea revealed high levels of free radicals such as superoxide anion, hydroxyl radical, and hydrogen peroxide in the cochlea of noise-exposed animals [21]. In a study conducted on animals subjected to acoustic trauma, it was observed that cochlear damage first started as small foci in the outer hair cells and spread to cover the entire segment of the organ of Corti with continued exposure [22]. According to a different study, focal lesions in the outer hair cells were more frequent than in the inner hair cells and increased with increasing sound levels [23]. In addition, a study conducted after 4 hours of acoustic trauma at 110, 115, and 120 dB found that karyorrhexis and karyopyknosis, which are indicators of apoptosis, were significantly higher especially in outer hair cells [24]. These findings provide a deeper understanding of the changes in the cochlea and are critical in efforts to develop potential therapeutic strategies against damage caused by acoustic trauma.

Acoustic trauma leads to the generation of acoustic energy in the cochlea, which is converted into neural signals and causes the release of free oxygen radicals. Endogenous defense systems exist to prevent the formation of these free radicals in the cochlea. These defense mechanisms include antioxidant systems, factors involved in signal transduction and proteins that respond to heat [25]. Strengthening these naturally occurring antioxidant mechanisms is effective in preventing or reducing hearing loss.

The effects of antioxidants on hearing due to acoustic trauma have been extensively studied in the literature. Protective effects of the combination of glutathione monoethylester (GEE) and R-N6-phenylisopropyladenosine (R-PIA) were observed against acoustic trauma applied to chinchillas [26]. In addition, the use of N-acetylcysteine (NAC) was observed to provide partial protection against impact noise trauma in rats, resulting in a significant improvement in hearing thresholds and a decrease in hair cell loss [27]. In addition, it was found that a combination of antioxidants (HPN-07 and NAC) given in the early post-explosion period reduced transient and permanent hearing loss and significantly reduced the loss of inner and outer hair cells [8]. Another study indicated that a combination of vitamins A, C, and E, and magnesium was highly effective in preventing noise-induced hearing loss and cell death [28]. All these studies show that antioxidants reduce damage in the cochlea after acoustic trauma and may be effective in protecting hearing functions.

Thymoquinone has been extensively studied in terms of its antioxidant properties. The key mechanism in the antioxidant activity of thymoquinone is its effective scavenging of harmful superoxide radicals [29]. Thymoquinone has been reported to increase blood flow in most tissues by enhancing metabolism, and, as a result, more oxygen is directed to cells affected by stress [30]. Thymoquinone has also been found to have significant anti-inflammatory effects in various diseases, including cardiovascular diseases such as atherosclerosis [31]. Studies on the effect of thymoquinone on hearing loss after acoustic trauma are limited. Aksoy et al. in their study on rats observed that thymoquinone improved post-traumatic hearing functions, especially in the days after trauma, and that there were improvements in auditory brainstem responses and OAE values [32]. Ogurlu et al. observed a significant improvement in the repair of auditory damage in rats treated with thymoquinone at a high dose (20 mg/kg) after acoustic trauma [33]. These results suggest that thymoquinone may be used as a potential treatment for the amelioration of hearing loss caused by acoustic trauma. In our study, no significant decrease was observed in OAE measurements made on the first day after acoustic trauma in the thymoquinone-treated group. This highlights the potential protective effects of thymoquinone. In particular, the attenuation of the effects of acoustic trauma on the first day of acoustic trauma in thymoquinone-treated rats provides an important indication that thymoquinone protects inner ear cells through oxidative stress-reducing and antioxidant mechanisms. Thymoquinone administration in the early stages of acoustic trauma is effective in minimizing potential trauma-induced damage and may contribute to the preservation of hearing functions. Our study is similar to the literature in that the transient threshold loss was better in the thymoquinone group compared to the thymoquinone-free group. However, unlike the two studies mentioned above, we observed positive results of thymoquinone use in the Thymoquinone group starting from the first day after acute trauma. The possible reasons for this difference may be as follows; firstly, administration of thymoquinone immediately after acoustic trauma may have increased the effect of antioxidant intervention in the early stages of damage and minimized the damage of the inner ear caused by oxidative stress. Secondly, the dosage of thymoquinone and the duration of treatment may have triggered a more effective recovery process than in other studies. Finally, environmental factors, such as the experimental setting and conditions of care of the animals, and biological characteristics, such as the genetic makeup of the animal model, may have also influenced our results by modulating the effects of thymoquinone. These factors may help us better understand the therapeutic potential of thymoquinone against hearing loss after acoustic trauma and may guide future research.

Limitations

Potential limitations of this study include the fact that the animal model used may not fully reflect the outcomes of acoustic trauma in humans. Research in rats may not fully model the complexity of human ear structure and mechanisms of hearing loss. Therefore, caution should be exercised in generalizing the findings to the human population. In addition, the dosage and frequency of administration used to evaluate the effects of thymoquinone require further research to determine optimal treatment protocols. The results obtained in the study may differ by testing different dosages and treatment durations. Another limitation may be the way and intensity of acoustic trauma during the study. Standardizing the sound level and duration of exposure could make the results more consistent. Furthermore, the study only tested noise exposure at a specific frequency, which may limit the validity of the results for other frequencies. Finally, the observation period of the study may not reflect the potential long-term effects of thymoquinone. Longer follow-up studies may provide more information about the sustainability and long-term effectiveness of the treatment. These factors are important elements that may influence the results of the study and should be considered in the interpretation of research results.

## Conclusions

This study examined the potential effects of thymoquinone treatment on hearing loss induced by acoustic trauma in rats. The results demonstrated that thymoquinone significantly reduced hearing loss and improved OAE values. In particular, early post-traumatic administration of thymoquinone treatment seems to contribute to the preservation of hearing function by reducing oxidative stress in the cochlea. However, further research is needed on the direct applicability of thymoquinone for the treatment of acoustic trauma in humans. Future studies are important in terms of optimization of different dosages and timing of administration of thymoquinone, development of treatment protocols, and increasing its efficacy. Furthermore, comprehensive studies on the long-term effects and potential side effects of thymoquinone should be conducted.
